# Retinol improves bovine embryonic development in vitro

**DOI:** 10.1186/1477-7827-2-83

**Published:** 2004-12-21

**Authors:** Tracy Livingston, Dawn Eberhardt, J Lannett Edwards, James Godkin

**Affiliations:** 1 Department of Biology, Georgetown College, Georgetown, KY 40324 USA; 2 AviGenics, Inc., 111 Riverbend Road, Athens, GA 30605 USA; 3 Department of Animal Science, University of Tennessee, Knoxville, TN 37996 USA

## Abstract

Retinoids are recognized as important regulators of vertebrate development, cell differentiation, and tissue function. Previous studies, performed both in vivo and in vitro, indicate that retinoids influence several reproductive events, including follicular development, oocyte maturation and early embryonic development. The present study evaluated in vitro effects of retinol addition to media containing maturing bovine oocytes and developing embryos in both a low oxygen atmosphere (7%) and under atmospheric oxygen conditions (20%). In the first experiment, abbatoir collected bovine oocytes were matured in the presence or absence of varying concentrations of retinol. After a 22–24 hour maturation period the oocytes were fertilized, denuded 18 hours later and cultured in a modified synthetic oviductal fluid (mSOF) in a humidified atmosphere at 38.5 degrees C, 5% CO2, 7% O2 and 88% N2. Cleavage rates did not differ among control and retinol-treated oocytes in all three experiments. Addition of 5 micromolar retinol to the maturation medium (IVM) tended (p < 0.07) to increase blastocyst formation (blastocyst/putative zygote; 26.1% +/- 2.2%) compared to the controls (21.9% +/- 1.9%). Further analysis revealed when blastocyst development rates fell below 20% in the control groups, 5 micromolar retinol treatment dramatically improved embryonic development, measured by blastocyst/putative zygote rate (14.4 +/- 2.1 vs 23.7 +/- 2.5; p < 0.02). The 5 micomolar retinol treatment also enhanced the blastocyst/cleaved rate by nearly 10% (23.7% vs 34.6%; p < 0.02). In the second and third experiments addition of 5 micromolar retinol to the embryo culture medium (IVC) under low oxygen conditions did not significantly improve cleavage or blastocyst rates, but 5 micromolar retinol significantly increased blastocyst development under 20% O2 conditions (p < 0.001). These studies demonstrate that supplementation of 5 micromolar retinol to the maturation medium may improve embryonic development of bovine oocytes indicated by their increased blastocyst rate. A significant improvement in the blastocyst development with the 5 micromolar retinol treatment under atmospheric conditions suggests a beneficial antioxidant effect during embryo culture.

## Background

Vitamin A is essential for reproduction, and deficiencies and excesses may result in embryonic loss and/or congenital defects [1]. Retinol (vitamin A alcohol) is the parent vitamin A compound and metabolites, analogs, and derivatives are known collectively as retinoids. Results from several studies, in a variety of species, have indicated that retinoid administration may function in very early events associated with reproductive success, including follicular development, oocyte maturation and early embryonic development. Retinol concentration in bovine follicular fluid was shown to be an indicator of follicular quality and was highest in healthy follicles, lowest in atretic follicles and highly correlated with estradiol concentrations [2,3]. Retinol or β-carotene administration has been shown to prevent fetal resorption in rats [4], increase the number of births in rabbits [5], and increase litter size in swine [6]. Retinol administration to ewes, in combination with superovulation followed by natural service was shown to improve the competence of resultant 1–4 cell and morula stage embryos, collected from the oviduct and uterus, respectively, to develop to the blastocyst stage when cultured in vitro [7]. In cattle, retinol injection improved the estimated quality of embryos collected from superovulated animals but did not increase the number recovered [8].

Retinol is transported systemically and intercellularly bound to retinol-binding protein (RBP). Cellular retinol-binding proteins (CRBP) and cellular retinoic acid-binding proteins function in intracellular vitamin A transport, metabolism and homeostasis [9]. All-*trans *and 9-*cis *retinoic acid (RA) are natural cellular metabolites of retinol and mediate biological activity through interaction with nuclear retinoic acid receptors (RAR) and retinoid X receptors (RXR), respectively. Ligand-bound RARs and RXRs influence transcription by interacting with response elements in the promoter regions of retinoid-regulated genes [10]. Within the ovary, RBP and CRBP are expressed in thecal and granulosa cells, and facilitate the transport of retinol from the blood into developing follicles [3]. Concentrations of RBP, and its ligand retinol, are highest in the follicular fluid of large preovulatory bovine ovarian follicles, compared to smaller and/or atretic follicles [3]. RBP synthesis and secretion increase in the oviduct and uterus coincident with the transport of the egg or embryo into these organs [11,12].

The cumulus oocyte complex (COC) may be a target for retinol, since the cells that nurture and communicate with the oocyte, contain transcripts and protein for several RARs and RXRs, RBP and retinaldehyde-2 dehydrogenase (RALDH-2) a metabolizing enzyme [13]. Bovine oocytes and embryos from the 2-cell to hatched blastocyst stage, also express transcripts for several RARs, RXRs, RBP and RALDH-2, and the inner cell mass and trophectoderm of blastocysts express immunoreactive protein for RAR and RXR [14]. It has been shown recently that addition of 9-*cis *RA to in vitro oocyte maturation medium affects trophectoderm differentiation, total cell number and inner cell mass-trophoblast cell ratios, following fertilization in cattle oocytes [15,16]. Together, these studies suggest that the reproductive tract delivers retinol to the oocyte and early embryo which possess key elements of retinoid metabolizing and signaling mechanisms; thus, influencing gene expression, differentiation, and development.

The mechanism by which retinol or retinoic acid administration influences oocyte maturation and positively impacts early embryonic development is not known and is the subject of much investigation. Retinoic acid may influence oocyte maturation through its effects on FSH or LH receptor expression as demonstrated in porcine [17] and rat [18] granulosa cells. Alternatively, it has been suggested that retinoic acid may increase mRNA quality and processing during maturation mediated by increases in polyadenylation [19]. Expression of several growth factors is influenced by RA [20]. Midkine [16], a member of the heparin-binding growth/differentiation family, is induced by RA and has been shown to improve bovine oocyte and embryonic developmental competence [21]. In addition, retinoids may promote development through participation in an endogenous oxidative-stress protection mechanism [22].

In the present study, we investigated the effects of retinol administration to in vitro matured oocytes, and cultured bovine embryos under atmospheric O_2 _and reduced O_2 _conditions. Results suggest beneficial effects of retinol administration during maturation especially to less competent oocytes, and improved development of embryos cultured under atmospheric oxygen conditions, indicating protection from oxidative stress.

## Materials and Methods

### Reagents and Media

All chemicals were purchased from Sigma Chemical Company, St. Louis, MO unless otherwise noted. Bovine oocyte collection medium (OCM) was composed of modified M199, 4.2 mM NaHCO_3_, 12 mM HEPES, and supplemented with 2 mM glutamine, 2% fetal bovine serum (FBS, BioWhittaker, Baltimore, MD), and penicillin/streptomycin (Specialty Media, Phillipsburg, NJ). Oocyte maturation medium (OMM) consisted of bicarbonate buffered TCM-199 supplemented with 50 μg/mL of gentamycin, purchased from Specialty Media, 5 μg/mL of FSH purchased from Vetrepharm Canada, Inc. (Ontario, Canada), 0.3 μg/mL of luteinizing hormone (LH) that was generously provided by the USDA, Beltsville, MD, 10% FBS, 0.2 μM sodium pyruvate and 2 mM glutamine. Modified Tyrode's Albumin Lactate Pyruvate (TALP) media used in sperm preparation (SP-TALP), removal of cumulus cells from oocytes (HEPES-TALP) and in vitro fertilization (IVF-TALP) were prepared as described by Parrish et al. [29]. In vitro culture (IVC) medium was a modified synthetic oviductal fluid (mSOF) [30] supplemented with 3 mg/mL of BSA, 0.6 mM sodium pyruvate, 2% (v/v) BME essential amino acids, 1% (v/v) MEM non-essential amino acids, and 100 μg/mL of penicillin and streptomycin.

All-*trans *retinol was dissolved in 100% ethanol, appropriate dilutions made, and aliquots were stored at -80°C until use. Retinol was prepared fresh each month and checked on a spectrophotometer for accuracy. The concentration of ethanol during maturation or culture was less than 0.1%.

### Collection and in vitro maturation (IVM) of oocytes

Ovaries from mature, cycling cattle were obtained from an abbatoir and pooled. Cumulus oocyte complexes (COCs) were quickly harvested by slicing follicles (2–5 mm) with a sterile surgical blade, and collecting them in OCM. Intact COCs with homogeneous ooplasm and two or more layers of cumulus cells were selected, washed, and approximately 50 were transferred to 500 μl of pre-equilibrated OMM, and matured for 22–23 hours in a 38.5°C incubator with an atmosphere of 5.0% CO_2_, ambient air, and saturated humidity.

### In vitro fertilization (IVF)

Fertilization (Day 0) was performed with combined semen from two bulls of proven fertility prepared according to the method by Parrish and coworkers [29]. Briefly, spermatozoa were washed in a discontinuous Percoll gradient (45%/90%) by depositing semen on top of the Percoll layers and centrifuged for 15 minutes at 960 *g*. The pellet was removed and resuspended in SP-TALP and centrifuged for 8 minutes at 460 *g*. After removal of the supernatant, the sperm sample was reconstituted in 500 μL of IVF-TALP for a final concentration of 1 × 10^6 ^spermatozoa/mL. The plate was incubated for 22 hours at 38.5°C in an atmosphere of 5.0% CO_2 _and ambient air with saturated humidity.

### In vitro culture (IVC)

Approximately 18 hours after fertilization putative zygotes were denuded of cumulus cells by vortexing in 500 μl of HEPES-TALP for four minutes (Day 1). Putative zygotes (approximately 35–40) were cultured in 500 μL of mSOF for eight days in a 38.5°C incubator in an atmosphere of 5% CO_2_, 7% O_2 _and 88% N_2 _(first and second experiments) with saturated humidity. The mSOF medium was changed every 48 hours. Cleavage was assessed on Day 3 and blastocyst rate was calculated on Day 8.

### Experimental Design

In the first experiment maturation medium alone was supplemented with all-*trans *retinol (0, 1.0, 5.0, or 10.0 μM) and embryos were allowed to develop under low oxygen conditions. In the second experiment all-*trans *retinol was added only to embryo culture medium (0, 1.0, 2.0, 5.0, or 10μM) on days 1, 3, 5, and 7, and the embryos developed in a low oxygen atmosphere. In the third experiment embryos were cultured under atmospheric oxygen conditions (air and 5% CO_2_) and all-*trans *retinol (0 or 5μM) was added to embryo culture medium on days 1, 3, 5, and 7.

### Data Analysis

Data were analyzed as an incomplete block design (experiments 1, 2, and 3), or a randomized block design (experiment 4), blocked on plate using mixed model procedures of SAS [31]. At least six replicates were completed for each experiment. Fisher's protected least significant differences were used for separating least square differences for experiments 1, 2, 3, and a two-tailed Student's T-test was performed on data from experiment 4. Least square means ± S.E.M. are expressed as the proportion of putative zygotes. All data were subjected to a normality test (Shapiro-Wilk, > 0.90) and were found to be normally distributed.

## Results

In the first experiment addition of 5μM retinol during IVM tended to improve (p < 0.07) embryonic development to the blastocyst stage, compared to controls (Table 1). The control blastocyst rate was 21.9% compared to 26.1% in 5μM retinol. Addition of 1μM retinol to the maturation medium did not appear to affect embryonic development compared to controls. Retinol (10μM) increased blastocyst development, although not significantly. Cleavage rates did not differ among the four maturation treatments.

**Table 1:** Effect of all-*trans *retinol addition to bovine oocyte maturation medium (mean ± S. E. M.). Embryos were cultured under low oxygen conditions.

Retinol concentration (μM)	Putative zygote (n)	Cleavage	Blastocyst/ putative zygote	Blastocyst/ cleaved
0	1095	66.7 ± 2.7	21.9 ± 1.9^a^	32.8 ± 2.2^a^
1.0	464	65.5 ± 3.9	20.4 ± 2.6^a^	31.7 ± 3.1^a^
5.0	1069	68.3 ± 3.2	26.1 ± 2.2^b^	37.1 ± 2.5^b^
10.0	508	70.1 ± 3.9	24.2 ± 2.7^ab^	33.8 ± 3.1^ab^

Values are listed as percentages. ^ab^Means in the same column with different superscripts approach significance (p < 0.07).

Further analysis of the maturation data (Table 2) revealed that when development to the blastocyst stage of controls was below 20%, 5μM retinol dramatically improved (p < 0.02) embryo development (14.4 % vs. 23.7%). When expressed as blastocyst/cleaved the 5μM retinol treatment also showed a significant improvement in blastocyst development (p < 0.02). Neither 1μM nor 10μM retinol treatment improved embryonic development when compared to those controls that did not achieve a 20% blastocyst rate.

**Table 2:** Effect of all-*trans *retinol addition to bovine oocyte maturation medium on embryo development among replicate groups where less than 20% of control embryos reached the blastocyst stage (mean ± S. E. M.).

Retinol concentration (μM)	Putative zygote (n)	Cleavage	Blastocyst/ putative zygote	Blastocyst/ cleaved
0	516	62.7 ± 3.9	14.4 ± 2.1^a^	23.7 ± 2.6^a^
1.0	185	60.0 ± 6.0	15.9 ± 3.4^ab^	26.1 ± 4.2^ab^
5.0	530	65.9 ± 4.6	23.7 ± 2.5^b^	34.6 ± 3.1^b^
10.0	183	63.3 ± 6.7	17.6 ± 3.8^ab^	26.7 ± 4.6^ab^

Values are listed as percentages. ^ab^Means in the same column with different superscripts were significantly different (p < 0.02).

Further experiments were conducted during IVC under both low and atmospheric oxygen tensions. Under low oxygen conditions concentrations of 1, 2, and 5μM retinol were not statistically different from controls, and 10μM was deleterious (Table 3). Preliminary dose-response studies were performed under atmospheric conditions (data not shown), and additional experiments were continued with the 5μM retinol treatment. Under atmospheric oxygen conditions the 5μM concentration significantly improved blastocyst development compared to controls (p < 0.001) (Table 4). Cleavage rates did not differ significantly among embryos treated with and without retinol during culture under low or high oxygen (Tables 3 &4). Fertilization rates did not differ significantly among all experiments (data not shown).

**Table 3:** Effect of all-*trans *retinol addition to the culture medium (mean ± S. E. M.). Embryos were cultured under low oxygen conditions.

Retinol concentration (μM)	Putative zygote (n)	Cleavage	Blastocyst/ putative zygote	Blastocyst/ cleaved
0	567	86.1 ± 2.5	26.5 ± 2.4^a^	30.7 ± 2.6^a^
1.0	312	84.7 ± 3.2	27.1 ± 3.2^ab^	32.1 ± 3.5^a^
2.0	414	85.3 ± 2.9	28.8 ± 2.8^a^	34.1 ± 3.1^a^
5.0	303	80.8 ± 3.2	20.2 ± 3.2^a^	25.4 ± 3.5^a^
10.0	388	81.2 ± 3.0	13.5 ± 3.0^c^	16.2 ± 3.3^b^

Values are listed as percentages. ^abc^Means in the same column with different superscripts were significantly different (p < 0.05).

**Table 4:** Effect of all-*trans *retinol addition to the culture medium (mean ± S. E. M.). Embryos were cultured under atmospheric oxygen conditions.

Retinol concentration (μM)	Putative zygote (n)	Cleavage	Blastocyst/ putative zygote	Blastocyst/ cleaved
0	400	73 ± 4.6	14 ± 2.3^a^	19.2 ± 3.2^a^
5.0	400	74 ± 1.5	28.8 ± 3.0^b^	38.9 ± 3.9^b^

Values are listed as percentages. ^ab^Means in the same column with different superscripts were significantly different (p < 0.001).

## Discussion

In the present study, over 3000 bovine oocytes were used to evaluate effects of retinol supplementation during IVM and IVC on embryonic development to the blastocyst stage. Retinol administration during the maturation period alone resulted in concentration-dependent effects. Whereas the presence of 1μM retinol had no effect on development, 5μM retinol tended to improve blastocyst rate of development, at the p < 0.07 level, compared to controls. At a concentration of 10μM, retinol did not significantly improve embryo development compared to controls. In preliminary studies, higher concentrations (100μM) were observed to be cytotoxic (data not shown). Similarly, exposure of bovine oocytes to low concentrations of 9-*cis *retinoic acid was shown to improve subsequent blastocyst development but high concentrations were detrimental [16].

A more striking effect on embryonic development (p < 0.02) was observed by supplementation of 5 μM retinol to groups of oocytes with reduced developmental competence in which development of control oocytes to blastocyst was less than 20%. These results indicate that retinol supplementation during maturation may not benefit oocytes competent to progress, but rather, it improves the viability of oocytes that are developmentally challenged. In support of this, we have shown previously that retinol supplementation during maturation improves developmental competence of bovine oocytes compromised by heat stress [32].

Since most transcription in the oocyte occurs prior to maturation during preovulatory development, in vitro culture deprives oocytes of much of this activity. Meiotic inhibitors have been used as a potential means of investigating regulation of oocyte transcription and mRNA processing in vitro [33]. Treatment of cumulus-enclosed oocytes with 9-cis RA during meiotic arrest was observed to improve cortical granule migration, increase subsequent blastocyst development and increase total cell number [34]. Gomez and co-workers [19] suggested that retinoid administration may improve mRNA quality based on the observation that 9-cis RA increased poly-(A) mRNA content in meiotically arrested oocytes. Poly-(A) mRNA content of oocytes treated with 9-cis RA or ethanol vehicle was greater in matured oocytes than in oocytes prematured in the presence of 9-cis RA and then matured.

Retinol supplementation of embryo culture medium dramatically improved development to the blastocyst stage (p < 0.001) when cultured in an atmosphere of approximately 20% O_2 _(air and 5% CO_2_) but not in an atmosphere of low O_2 _(7% O_2_, 5% CO_2 _and 88% N_2_). The present study, and all previous in vitro studies demonstrating a positive effect of retinoid administered during maturation, were performed in an atmosphere of approximately 20% O_2 _[15,16,34], a practice common to most laboratories. Together, these data indicate that retinoids may protect embryos from oxidative damage, which has been identified as a leading cause of embryonic wastage, especially in vitro [22].

Mammalian cells, including the oocyte and those of the early embryo, have evolved several mechanisms to protect against ROS damage and maintain appropriate balances in REDOX reactions. Antioxidants present in the oocyte, embryo and/or its environment include vitamins A (retinol), C and E, pyruvate, glutathione (GSH), hypotaurine, taurine, and cysteamine [22]. Antioxidant enzymes produced by oocytes and embryos include, copper, zinc superoxide dismutase (Cu, Zn-SOD), manganese-SOD (Mn-SOD), glutathione peroxidase (GPX), glutamyl cysteine synthase (GCS), glutathione reductase (GR), catalase and others [22]. Lonergan and co-workers [36] have shown that expression of several antioxidant enzymes are up-regulated during in vitro oocyte maturation compared to in vivo maturation indicating that the former environment creates oxidative stress and oocytes respond by activating internal defense mechanisms. Addition of antioxidants to culture medium or culture of embryos in an atmosphere of reduced O_2 _has been demonstrated to be beneficial to in vitro survival of embryos from a variety of species [22].

Retinoids participate in a biological antioxidant network, and have been implicated as important regulators of redox signaling pathways [23,24]. Carotenoids and retinol can quench single oxygen molecules and interact with other antioxidant compounds [23]. Retinoic acid has been shown to protect against oxidative stress-induced apoptosis by inhibition of the c-jun N-terminal kinase (JNK) activator protein 1 (AP-1) pathway in glomerular [26] and mesangial cells [37]. In addition, anti-apoptotic effects of RA were mediated by both nuclear receptor-dependent and independent pathways [37].

Retinoids may also protect against oxidative damage by maintaining adequate endogenous levels of antioxidant compounds and enzymes. Glutathione is the major non-protein sulphydryl compound found in mammalian cells responsible for strong basal ROS scavenging activity [35]. Maintenance of adequate GSH levels is essential for oocyte maturation, fertilization and embryonic development [22]. Retinoic acid inhibited staurosporine-induced GSH depletion in neuronal cells, preventing oxidative damage and apoptosis [25]. A retinoic acid response element (RARE) has been identified in the promoter region of a specific isoform of glutathione S-transferase-pi (GSTp) in glioblastoma cells [38] and GPX2 [28], an enzyme necessary for the conversion and utilization of GSH. RA has also been shown to significantly increase survival, reduce ROS content and increase protein levels of Cu-Zn SOD and Mn-SOD in neuronal cells treated with staurosporine [27]. Recently, microarray analysis revealed that three genes which encode enzymes involved in GSH synthesis and utilization were RXRα-target genes in mouse liver [39]. The same study showed that in hepatocytes of RXRα-deficient mice there was a significant reduction in GSH synthesis rate and GSH content [39]. Together, these data provide strong evidence that in several cell systems, retinoids support and improve endogenous antioxidant defense mechanisms.

## Conclusions

Results from the present study indicate that retinol administration during in vitro maturation particularly improved embryonic development in those oocytes that may have been developmentally compromised. Moreover, retinol addition during in vitro culture, under atmospheric conditions, also improved embryonic development compared to those embryos incubated in a 7% oxygen atmosphere. The mechanisms by which retinoids affect the developmental capacity of oocytes and early embryos may include modulation of expression of growth factors and other developmental genes, improving mRNA quality, and direct and/or indirect affects on antioxidant defense mechanisms.

## Authors' contributions

**TL **Performed the experiments under low oxygen conditions, helped to coordinate experiments, and drafted the manuscript.

**DE **Performed the experiments under high oxygen conditions and helped to coordinate experiments.

**JE **Coordinated and assisted in the experimental design.

**JG **Conceived of and coordinated the experiments and drafted the manuscript.
